# Antibiotic Resistance in *Staphylococcus aureus* and Coagulase Negative Staphylococci Isolated from Goats with Subclinical Mastitis

**DOI:** 10.4061/2010/517060

**Published:** 2010-02-03

**Authors:** Salvatore Virdis, Christian Scarano, Francesca Cossu, Vincenzo Spanu, Carlo Spanu, Enrico Pietro Luigi De Santis

**Affiliations:** Dipartimento di Biologia Animale, Università di Sassari, via Vienna 2, 07100 Sassari, Italy

## Abstract

Antimicrobial resistance patterns and gene coding for methicillin resistance (*mecA*) were determined in 25 *S. aureus* and 75 Coagulase Negative Staphylococci (CNS) strains isolates from half-udder milk samples collected from goats with subclinical mastitis. Fourteen (56.0%) *S. aureus* and thirty-one (41.3%) CNS isolates were resistant to one or more antimicrobial agents. *S. aureus* showed the highest resistance rate against kanamycin (28.0%), oxytetracycline (16.0%), and ampicillin (12.0%). The CNS tested were more frequently resistant to ampicillin (36.0%) and kanamycin (6.7%). Multiple antimicrobial resistance was observed in eight isolates, and one *Staphylococcus epidermidis* was found to be resistant to six antibiotics. The *mecA* gene was not found in any of the tested isolates. Single resistance against *β*-lactamics or aminoglicosides is the most common trait observed while multiresistance is less frequent.

## 1. Introduction

Raw goat's milk can be a potential source of antibiotic-resistant pathogens of animal, human, and environmental origin. The microorganisms which contaminate raw milk may originate from the farm environment or from the goats and include the etiological agents responsible for clinical and Subclinical Mastitis (SCM). 

 In dairy goats with SCM, Coagulase Negative Staphylococci (CNS) make up 44.7% to 95.9% of the isolated pathogens from milk samples, and *S. aureus*, which is usually considered to have the greater pathogenicity, accounts from 4.1% to 18.0% of SCM agents [1]. The average prevalence of SCM in dairy goat's farms is between 20.0%–35.0% and results in significant economic losses due to reduction in milk production and poor milk quality [1]. 

 The intramammary administration of antibiotics used on farms has increased, as it was proved to be effective for treating SCM in dry small ruminants [2, 3]. The efficacy of intramammary antibiotic treatment could be compromised by staphylococci that produce biofilms in the udder.

The widespread use of antibiotics on dairy farms, could lead to the selection and to the emergence of antibiotic-resistant bacterial strains [4]. 

 Most published scientific research papers had for object the antimicrobial resistance of intramammary infection pathogens isolated from raw bovine milk. The detection in raw milk of multiple resistant strains, and especially methicillin resistant *S. aureus* (MRSA) and CNS (MRCNS) strains, is regarded as an issue of great concern for their potential spread through the dairy food chain. In small ruminants, the spread of MRSA strains is controversial. MRSA strains are characterized by the presence of the *mecA* gene encoding low-affinity penicillin binding protein (PBP2′), which mediates resistance to all classes of *β*-lactam antibiotics [5]. Few studies have reported that *S. aureus* strains, isolated from ewes milk affected by SCM, did not carry the *mecA* gene [6], or that, the few strains that were resistant to methicillin (oxacillin), probably were of human origin [7]. Instead, other studies showed how MRCNS strains can be isolated from goats milk with SCM [8]. 

 Antibiotic resistance pattern for staphylococci isolated from SCM refers mainly to cattle, and little is known about dairy goats [8–12]. Studies on antimicrobials susceptibility of these pathogens [13] have been mainly conducted using the agar disc diffusion method of Bauer et al. The broth microdilution and the agar dilution methods [14] instead allow the evaluation of the minimum inhibitory concentrations (MIC). 

 The evaluation of the antimicrobial susceptibility of *Staphylococcus *spp. isolated from goats with SCM is of great interest for clinical purposes in order to decide which antibiotics should be administered, as well as, for monitoring the spread of multiple resistant strains on farms. The current study seeks to support the sparse literature on antibiotic resistance of *S. aureus* and CNS isolated from goats with SCM. MICs and in vitro susceptibilities to ten antibiotics used in the medical and veterinary fields were determined against 25 *S. aureus* and 75 CNS strains. On these strains, the frequencies of single and multiple antibiotic-resistance were also evaluated.

## 2. Materials and Methods

### 2.1. Dairy Farms

The milk samples were collected from eight goats flocks in the island of Sardinia (Italy) where mainly Sarda and Sarda-Maltese breeds were reared. The rearing system was extensive and the animals were hand-milked. The goats, fed on bushes and grass, occasionally were supplemented with concentrates. The mean flock size was 187.5 ± 25.2 (mean ± SD) with a range from 160 to 234. Goats were not treated with systemic or intramammary antimicrobial agents during the lactation previous to the enrollment in this study nor during the dry period.

### 2.2. Sampling

A clinical examination of half-udders was conducted in order to exclude animals with signs of clinical mastitis [15]. A total of 3,000 half-udder milk samples (1,500 goats) were collected in a single sampling time in each of the eight flocks during the early lactation period (from January to April of 2006). The first few streams of foremilk were discarded, and duplicate half-udder milk samples were aseptically collected into sterile tubes after cleaning and disinfection of each teat end. One sample (10 mL) was used for bacteriological analysis and the other one (50 mL) was added with bronopol (2-bromo-2-nitropropane-1,3-diol) and analysed for Somatic Cell Count (SCC). The milk samples were stored at +4°C, and bacteriological and SCC examinations were carried out within 6 and 72 hours after sampling.

### 2.3. Bacteriological Analysis and Isolation Procedures

Each half-udders milk sample was mixed by inversion and 10 *μ*L were then inoculated onto 5.0% Sheep Blood Agar (SBA) plates. The SBA plates were then incubated aerobically at +37°C and examined after 48 and 72 hours. A significant bacterial count was considered when a growth of ≥500 identical cfu/mL was detected. Bacterial strains were isolated on Brain Heart Infusion agar (BHI, Oxoid, Basingstoke, UK), and identified using routinary microbiological procedures such as colony morphology, microscopic characteristics and Gram staining, hemolysis pattern on SBA, catalase and oxidase reactions. The strains were identified using API ID32 STAPH system (bioMérieux, Lyon, France) and the strips were read by the mini API instrument and associated software V 1.5.2 (bioMérieux, Lyon, France). The strains were then frozen at −80°C in BHI broth (Oxoid, Basingstoke, UK) with 15.0% glycerol.

### 2.4. Bacterial Characterization

Among the isolated microorganisms 100 *Staphylococcus* spp. strains were selected, 25 *S. aureus* (all isolates) and 75 CNS (randomly). Each strain was analysed for: haemolysis pattern on SBA at 5.0%, lecithinase activity on Baird Parker Medium (BPM, Oxoid, Basingstoke, UK), supplemented with Egg Yolk Tellurite Emulsion (EYTE) at 5.0% (Oxoid, Basingstoke, UK); thermostable DNase (TDNase, Biolife, MI, Italy) tested on toluidine blue-DNA agar plates [16], free coagulase (Coagulase plasma - EDTA, bioMérieux, Lyon, France) and bound coagulase (clumping factor) production (Staphylase test, bioMérieux, Lyon, France), following the manufacturer's instructions.

### 2.5. Somatic Cell Count (SCC)

The SCC was determined by the fluoro-opto-electronic cell counting method according to the FIL-IDF no. 148 (1995) method C [17], using a Fossomatic 5000 (Foss Electric, Hillerød, Denmark).

### 2.6. Definition of Subclinical Mastitis

For the purpose of this work, a mammary gland was considered as affected by subclinical mastitis when having no clinical signs or abnormal milk, in which ≥500 cfu/mL were isolated and a somatic cell count was ≥300,000 cells/mL [18].

### 2.7. Antimicrobial Agents and Minimal Inhibitory Concentration (MIC)

On each strain the MICs of ten antibiotics used in human and veterinary medicines were determined. The antibiotics tested were ampicillin (AMP), cephalothin (KF), cefoperazone (CFP), ceftriaxone (CRO), cloxacillin (OB), kanamycin (K), novobiocin (NV), ofloxacin (OFX), oxytetracycline (OT), and vancomycin (VA). The MICs were determined by the broth microdilution method [14, 22] using cation-adjusted Mueller-Hinton broth (CAMHB, Oxoid, Basingstoke, UK). Each antimicrobial agent, in powder form (Sigma-Aldrich-Fluka, MI, Italy), was weighed and dissolved in an appropriate solvent [14, 22], thus obtaining a stock solution (2,560 *μ*g/mL). Stock solutions were stored at −80°C until used. From each stock solution, 12 serial twofold working dilutions in deionized water (only for AMP, the diluents were phosphate buffer, pH 6.0, 0.1 mol/L) was prepared according to CLSI standard protocols, and the antimicrobial agent final concentrations in each microplate ranged between 0.06 and 128 *μ*g/mL. Each strain stored at −80°C until testing were subcultured twice on BHI agar (Oxoid, Basingstoke, UK) before inoculum preparation. Two or more identical colonies were picked from BHI plates after overnight incubation and suspended in saline solution (0.85% w/v) to match a McFarland 0.5 turbidity standard, using an inoculum reader (portable photometer Densimat, bioMérieux, Lyon, France). Each suspension was further diluted 1 : 100 in CAMHB in order to achieve the adequate inoculum concentration (≃10^6^ cfu/mL). Fifty microliters of the final suspension were inoculated into the wells of microtiter plates, which also contained 50 *μ*L of the antimicrobial agent, so that the final inoculum density on test plates contained ≃5 x 10^5^ cfu/mL in each well. Reference strains, *Staphylococcus aureus* ATCC 29213 and *Enterococcus faecalis* ATCC 29212, were used for quality control. Each microplate was then incubated at 35 ± 2°C under aerobic environment for 20 hours. The susceptibility of each strain to the antimicrobial agents was then defined by comparing the results to those of the breakpoint values [19–21]. The MICs range and mode, MIC_50_ and MIC_90_ of each antimicrobial agent were also determined.

### 2.8. Detection of mecA Gene

Genomic DNA used as target for polymerase chain reaction (PCR) assay was extracted using the following procedure: strains grown in BHI broth at +37°C overnight were centrifuged (10 minutes at 3,000 × g) and resuspended in 500 *μ*L of Tris-EDTA buffer saline (Tris base 10 mM + EDTA 1 mM). The suspension was added with 10 *μ*L of a 1.5 mg/mL lysostaphin solution (Sigma-Aldirich, MI, Italy) and incubated for 1 hour at +37°C. Then, 5 *μ*L of a 20 mg/mL proteinase K solution (Eurobio, Sarreguemines, France) were added and the incubation was continued at +50°C for 60 minutes. An equal volume of phenol/chloroform/isoamyl alcohol (25 : 24 : 1) was added and mixed by inversion. After centrifuging (15 minutes at 10,000 × g), the upper layer was collected and 500 *μ*L of chloroform-isoamyl alcohol (24 : 1) solution were added. The mixture was centrifuged again (15 minutes at 10,000 × g) and the upper aqueous phase was transferred into a new tube. A volume of 800–1,000 *μ*L of refrigerated absolute ethanol was added and gently mixed until DNA precipitation. DNA was resuspended in 100 *μ*L of sterile deionized water. The DNA concentration was estimated spectrophotometrically. The *mecA* gene coding for methicillin resistance was detected by PCR as previously described [23]. The primers used for the detection of the *mecA* gene were AAAATCGATGGTAAAGGTTGGC (forward) and AGTTCTGCAGTACCGGATTTGC (reverse). *S. aureus* HT 2004 0874 reference strain was used as positive control [24].

## 3. Results

### 3.1. Isolates

Bacteriological cultures were positive in 469 (15.6%) out of 3,000 half-udder milk samples. The intramammary infection rates in the eight flocks were, respectively, of 16.7% (range 15.0%–18.6%) and 14.6% (range 12.6%–17.4%) for the left and right half-udders. Bacterial strains isolated from milk samples were 415 CNS (88.5%), 25 *S. aureus* (5.3%), 4 *Micrococcus* spp. (0.9%), while 21 (4.5%) were identified as belonging to other species (*Bacillus* spp, *Enterococcus* spp. and Gram-Negative Bacilli), and 4 (0.9%) could not be identified by API system. The CNS strains were identified as follows: 187 *S. caprae* (45.1%), 64 *S. warneri* (15.4%), 41 *S. simulans* (9.9%), 31 *S. chromogenes* (7.5%), 16 *S. epidermidis* (3.9%), 9 *S. xylosus* (2.2%), 8 *S. haemolyticus* (1.9%), 7 *S. capitis *(1.7%), 6 *S. cohnii* (1.4%), 6 *S. lugdunensis* (1.4%), 5 *S. equorum* (1.2%), 5 *S. hominis* (1.2%), and 30 *Staphylococcus* spp. (7.2%). The mean SCC of bacteriological positive samples was 6.3 Log_10_ cells/mL, while in the negative ones it was 5.7 Log_10_ cells/mL. The mean SCC was greater in milk samples positive for *S. aureus* (6.8 Log_10_ cells/mL) when compared to those positive for CNS (6.4 Log_10_ cells/mL).

### 3.2. Selected Isolates

A total of 100 isolates were selected for antimicrobial agent susceptibility testing as follows: *S. aureus *(25), *S. caprae* (25), *S. warneri* (16), *S. simulans* (15), *S. chromogenes* (7), *S. epidermidis* (6), *S. equorum* (2), *S. cohnii* (1), *S. haemolyticus* (1), *S. lugdunensis* (1), and *S. xylosus* (1). The *S. aureus* strains were isolated from five out of eight flocks ranging between 2 and 8 for each one. Fifteen (60.0%) of the *S. aureus* strains showed hemolytic activity and among these, 5 (20.0%) produced *α*-hemolysin*,* 8 (32.0%)  *β*-hemolysin and 2 (8.0%) were *α* and *β*-hemolytic. Thirteen (17.3%) of the CNS strains showed a weak hemolytic activity on SBA. Free coagulase was produced by all the *S. aureus* strains tested, while three of these (12.0%) were negative to the bound coagulase test. All the CNS strains were found to be negative for the free coagulase and only one *S. lugdunensis* strain (1.3%) was clumping factor positive. TDNase was produced by all the *S. aureus* strains and by 17 (22.7%) of the CNS strains. Lecithinase was produced by 12 *S. aureus* (48.0%) and 27 CNS strains (36.0%).

### 3.3. Antimicrobials Susceptibility

The MICs of antibiotics and the susceptibility of *S. aureus* and CNS strains isolated from goats with SCM are shown Tables 1-2. Fourteen (56.0%) of 25 *S. aureus* and thirty-one (41.3%) out of 75 CNS strains were resistant to one or more antimicrobials. The susceptibility of *S. aureus* was 92.0% or greater for seven out of ten antibiotics tested but was lower for kanamycin (60.0%), oxytetracycline (84.0%), and ampicillin (88.0%). The susceptibility of CNS was between 94.0%–100.0% for eight antimicrobials, but was somewhat lower for ampicillin (64.0%) and kanamycin (78.7%). *Staphylococcus* spp. isolates showed a poor susceptibility to AMP. The MIC_90_ (1–4 *μ*g/mL) of this antibiotic was higher than the breakpoint value for susceptibility. Among CNS resistant to AMP, the most prevalent species were *S. caprae* (37.0%) and *S. chromogenes* (22.2%), while only 12.0% of the *S. aureus* strains were resistant. On the other hand, almost all staphylococci (98.0%) were susceptible to OB and only 2 out of 75 (2.7%) of the CNS were resistant. Cephalosporins showed high activity against *Staphylococcus* spp. isolates. The percentages of susceptible staphylococci were 98.0%, 99.0% and 96.0% for KF, CFP, and CRO, respectively. The *mecA* gene was not found in any of the tested isolates. For K a low susceptibility of the isolates (74.0%) was recorded. The MIC_90_ of K against the CNS and *S. aureus* strains (32–≥128 *μ*g/mL) was higher than the reference breakpoint for antimicrobial susceptibility. A different susceptibility to OT was observed in CNS isolates (94.7%) when compared to that of *S. aureus *(84.0%). The MIC_90_ of OFX for both *S. aureus* and CNS was comparable with the reference breakpoint for antimicrobial susceptibility (≤1 *μ*g/mL). All the tested staphylococci were susceptible to VA (100.0%), and 98.0% of these to NV. The NV resistant strains belonged to the *S. chonii* (*n* = 1) and *S. xylosus* (*n* = 1) species. The isolates which were resistant to two or more antimicrobial agents are shown in Table 3. It is remarkable that one *S. epidermidis *strain was resistant to six different antibiotics (AMP, CRO, K, OB, OFX, and OT).

## 4. Discussion

In the present study, the average prevalence of SCM was 15.6%, which is within the range (6.5%–67.0%) reported in previous studies carried out on dairy goat farms [1]. Staphylococci, which made up 88.5% of the isolated microorganisms in this study, are the most common pathogens associated with SCM in dairy goats [25]. 

 AMP was less effective than the other *β*-lactam antimicrobial agents. The susceptibility of the isolates against AMP was within the range reported by other authors [9, 26], even if Moroni et al. [27], have found a markedly greater prevalence of AMP resistant *S. aureus* (67.9%). These findings are consistent with AMP sensitivity to the penicillinases, frequently produced by *Staphyloccus* spp. and particularly by CNS strains. OB, a penicilinase-resistant penicillin (PRP), was very effective in vitro. Two CNS resistant to OB were simultaneously resistant to other *β*-lactamic antimicrobials, such as AMP (*n* = 1) and AMP-CRO (*n* = 1). The Cephalosporins showed a strong activity with regard to staphylococci. The MIC_90_ values of CFP ranged between 0.25 and 8 *μ*g/mL, that were comparable (1.87–3.75 *μ*g/mL) to those found by Moroni et al [12]. Nevertheless, a low susceptibility to CFP was previously found in some CNS strains isolated from goats with SCM, particularly with regard to *S. chromogenes*, *S. warneri*, *S. simulans*, and *S. kloosii *[11]. Other authors found that the susceptibility to KF of CNS isolated in goats with SCM was between 86.0% and 100.0% [10, 28], that is, comparable with the results (98.7%) obtained in this study. The *mecA* gene was not found in any of the strains tested, in agreement with the results of a previous study carried out on *S. aureus* strains isolated from sheep with SCM [6]. The results of the present study confirm that methicillin-resistant staphylococci prevalence is still low in ruminants as observed in previous research [29]. The finding of some *mecA*-negative isolates which were phenotipically resistant to *β*-lactam antimicrobial agents could be related to a less common type of resistance due to either overproduction of *β*-lactamase or the presence of altered Penicillin Binding Protein (PBP) not related to 2a or 2′ [30]. The susceptibility to OT was lower in *S. aureus* (84.0%) than in CNS (94.7%). In previous studies, a number of authors have observed a marked variability in the susceptibility of both these microorganisms to tetracycline, as it ranged between 10.0% and 100.0% [8, 10, 12, 28]. The susceptibility of CNS against NV was 97.3% and this peculiarity is of interest in the taxonomy for bacterial typing since it is also well related with pathogenic activity [31]. All staphylococci were sensitive to VA, thus confirming the results of other authors [10, 32]. The VA breakpoint value was recently reduced from ≤4 *μ*g/mL to ≤2 *μ*g/mL in testing the susceptibility of bacterial strains isolated from humans [20]. Some staphylococci isolated from milk samples taken from goats with SCM showed MIC values of 4 *μ*g/mL. When these strains are transferred from animals to humans, they could increase the spreading of vancomycin-intermediate *S. aureus* (VISA) strains. Indeed, comparing the MIC values of VA that we found with the breakpoint actually used for human origin strains, a relevant percentage of *S. aureus* (16.0%) and CNS (13.3%) would be classified as intermediate.

## 5. Conclusion

This study confirms that staphylococci are the most common pathogens associated with SCM in dairy goats. As expected, the bacteriological positive milk samples had a SCC greater than the negative ones. Over 40.0% of the tested staphylococci were resistant to at least one antimicrobial agent. Single resistance against *β*-lactamics or aminoglicosides is the most common trait observed. Multiple antibiotic resistance was found in few of the tested Staphylococci strains, mainly in CNS. Although the methicillin-resistant staphylococci represent the most important pathogens responsible of humans severe hospital-acquired infections, the absence of *mecA* gene and the low prevalence of single and multiple antibiotic resistance suggest that SCM in goats does not play a significant role in the spreading of multiresistant staphylococci and it does not represent a great public health concern.

## Figures and Tables

**Table 1:** MIC (*μ*g/mL) of antimicrobials against *S*.  *a* *u* *r* *e* *u* *s* and *C* *N* *S* strains isolated from goats subclinical mastitis.

	*S. aureus*				*CNS*			
Antimicrobial agents	MIC_50_	MIC_90_	mode	range	MIC_50_	MIC_90_	mode	range
*Ampicillin* ^(*a*)^	0.12	1.0	≤0.06	≤0.06–16.0	0.12	4.0	≤0.06	≤0.06–16.0
*Cefoperazone* ^(b)^	2.0	8.0	1.0	1.0–8.0	2.0	4.0	2.0	≤0.06–32.0
*Ceftriaxone* ^(b)^	4.0	8.0	4.0	1.0–16.0	2.0	8.0	2.0	≤0.06–≥128
*Cephalothin* ^(a)^	0.25	0.25	0.25	≤0.06–≥128	0.25	0.25	0.25	≤0.06–16.0
*Cloxacillin* ^(a)^	0.25	0.5	0.25	1.0–0.12	0.5	1.0	0.5	≤0.06–≥128
*Kanamycin* ^(a)^	16.0	≥128	32.0	1.0–≥128	8.0	32.0	16.0	≤0.06–≥128
*Novobiocin* ^(c)^	0.12	0.5	≤0.06	≤0.06–0.5	0.12	0.5	≤0.06	≤0.06–32.0
*Ofloxacin* ^(b)^	0.5	1.0	0.5	0.25–2.0	0.5	1.0	0.5	≤0.06–32.0
*Oxytetracycline* ^(a)^	0.25	64.0	0.25	0.12–≥128	0.25	1.0	0.25	≤0.06–≥128
*Vancomycin* ^(a)^	2.0	4.0	2.0	0.5–4.0	2.0	4.0	2.0	≤0.06–4.0

^(a)^Breakpoints NCCLS, [19]; ^(b)^= breakpoints CLSI, [20]; ^(c)^= breakpoint [21].

**Table 2:** Antimicrobial susceptibility of *S*.  *a* *u* *r* *e* *u* *s* and *C* *N* *S* strains isolated from goats subclinical mastitis.

		*S. aureus*						*CNS*					
Antimicrobial	MIC (*μ*g/mL)	Susceptible		Intermediate		Resistant		Susceptible		Intermediate		Resistant	
agents	breakpoints	*n*	%	*n*	%	*n*	%	*n*	%	*n*	%	*n*	%
*Ampicillin* ^(a)^	≤0.25–≥0.5	22	*88.0*	–	–	3	12.0	48	*64.0*	–	–	27	*36.0*
*Cefoperazone* ^(b)^	≤16.0–≥64.0	25	*100.0*	–	–	–	–	74	*98.7*	1	*1.3*	–	–
*Ceftriaxone* ^(b)^	≤8.0–≥64.0	23	*92.0*	2	*8.0*	–	–	73	*97.3*	1	*1.3*	1	*1.3*
*Cephalothin* ^(a)^	≤8.0–≥32.0	24	*96.0*	–	–	1	4.0	74	*98.7*	1	*1.3*	–	–
*Cloxacillin* ^(a)^	≤2.0–≥4.0	25	*100.0*	*—*	*—*	–	–	73	*97.3*	*—*	*—*	2	*2.7*
*Kanamycin* ^(a)^	≤16.0–≥64.0	15	*60.0*	3	*12.0*	7	28.0	59	*78.7*	11	*14.7*	5	*6.7*
*Novobiocin* ^(c)^	≤4.0	25	*100.0*	*—*	*—*	–	–	73	*97.3*	*—*	*—*	2	*2.7*
*Ofloxacin* ^(b)^	≤1.0–≥4.0	23	*92.0*	2	*8.0*	–	–	71	*94.7*	1	*1.3*	3	*4.0*
*Oxytetracycline* ^(a)^	≤4.0–≥16.0	21	*84.0*	–	–	4	16.0	71	*94.7*	–	–	4	*5.3*
*Vancomycin* ^(a)^	≤4.0–≥32.0	25	*100.0*	–	–	–	–	75	*100.0*	–	–	–	–

^(a)^Breakpoints NCCLS, [19];  ^(b)^= breakpoints CLSI, [20];  ^(c)^= breakpoint [21].

**Table 3:** Staphylococcus spp. with multiple resistance to antibiotics (*μ*g/mL).

	*n* (%)	AMP^(a)^	CRO^(b)^	K^(a)^	OB^(a)^	OFX^(b)^	OT^(a)^
*S. aureus*	1 (4)	—	—	≥128	—	—	≥128.0
*S. caprae*	3 (12)	0.25–2–8	—	—	—	4	≥128.0
*S. epidermidis*	2 (33)	8.0	≥128.0	≥128.0–64.0	≥128.0	8.0–32.0	≥128.0
*S. simulans*	1 (7)	0.5	—	—	16.0	—	—
*S. warneri*	1 (6)	1.0	—	—	—	—	≥128.0

AMP = Ampicillin; CFP = Cefoperazone; CRO = Ceftriaxone; KF = Cephalothin; K = Kanamycin; NV = Novobiocina; OB = Cloxacillin; OFX = Ofloxacin; OT = oxytetracycline; VA = Vancomycin. ^(a)^breakpoints NCCLS, [19]; ^(b)^breakpoints CLSI, [20].

## References

[1] Contreras A, Luengo C, Sanchez A, Corrales JC (2003). The role of intramammary pathogens in dairy goats. *Livestock Production Science*.

[2] Poutrel B, De Crémoux R, Ducelliez M, Verneau D (1997). Control of intramammary infections in goats: impact on somatic cell counts. *Journal of Animal Science*.

[3] De Santis EPL, Mencarelli A, Nieddu MP (2001). Efficacia della somministrazione endomammaria di cloxacillina benzatina per il trattamento delle infezioni intramammarie dell’ovino nel corso dell’asciutta. *Large Animals Review*.

[4] Walther B, Friedrich AW, Brunnberg L, Wieler LH, Lübke-Becker A (2006). Methicillin-resistant *Staphylococcus aureus* (MRSA) in veterinary medicine: a “new emerging pathogen”?. *Berliner und Munchener Tierarztliche Wochenschrift*.

[5] Schnellmann C, Gerber V, Rossano A (2006). Presence of new *mecA* and mph(C) variants conferring antibiotic resistance in *Staphylococcus* spp. isolated from the skin of horses before and after clinic admission. *Journal of Clinical Microbiology*.

[6] De Santis EPL, Mureddu A, Mazzette R, Scarano C, Bes M Detection of enterotoxins and TSST-1 genes in *S. aureus* isolates from sheep subclinical mastitis.

[7] Vautor E, Carsenti-Dellamonica H, Sabah M, Mancini G, Pépin M, Dellamonica P (2007). Characterization of *Staphylococcus aureus* isolates recovered from dairy sheep farms (*agr* group, adherence, slime, resistance to antibiotics). *Small Ruminant Research*.

[8] Bochev I, Russenova N (2005). Resistance of *Staphylococcus* spp strains isolated from goat with sub clinical mastitis. *Bulgarian Journal of Veterinary Medicine*.

[9] Adegoke GO, Ojo MO (1982). Biochemical characterization of staphylococci isolated from goats. *Veterinary Microbiology*.

[10] Bedidi-Madani N, Richard Y, Borges E, Lerondelle C (1992). Identification and susceptibility to antibiotics of coagulase negative staphylococci isolated from goat milk. *Revue de Médecine Vétérinaire*.

[11] Moroni P, Vellere F, Antonini M, Pisoni G, Ruffo G, Carli S (2004). Antibiotic susceptibility of coagulase-negative staphylococci isolated from goats’ milk. *International Journal of Antimicrobial Agents*.

[12] Moroni P, Pisoni G, Antonini M (2005). Subclinical mastitis and antimicrobial susceptibility of *Staphylococcus caprae* and *Staphylococcus epidermidis* isolated from two Italian goat herds. *Journal of Dairy Science*.

[13] Wray C, Gnanou J-C (2000). Antibiotic resistance monitoring in bacteria of animal origin: analysis of national monitoring programmes. *International Journal of Antimicrobial Agents*.

[14] Clinical and Laboratory Standards Institute (2006). *Methods for Dilution Antimicrobial Susceptibility Tests for Bacteria That Grow Aerobically; Approved Standard*.

[15] Donovan GA, Risco CA, Shearer JK (1992). Assessment of the mammary system. *The Veterinary Clinics of North America*.

[16] Lachica RV, Genigeorgis C, Hoeprich PD (1971). Metachromatic agar-diffusion methods for detecting staphylococcal nuclease activity. *Applied Microbiology*.

[17] FIL-IDF (1995). *Enumeration of Somatic Cells*.

[18] Ribeiro MG, Megid J, Meira DR, Lara VM, Cortez A (1999). Mastite caprina. Estudo microbiológico, físico-químico e do diagnóstico através de provas indiretas. *Biológico*.

[19] National Committee for Clinical Laboratory Standards (2002). *Performance Standards for Antimicrobial Disk and Dilution Susceptibility Tests for Bacteria Isolated from Animals; Approved Standard*.

[20] Clinical and Laboratory Standards Institute (2006). *Performance Standards for Antimicrobial Susceptibility Testing; Sixteenth Informational Supplement*.

[21] Thornsberry C, Burton PJ, Yee YC, Watts JL, Yancey RJ (1997). The activity of a combination of penicillin and novobiocin against bovine mastitis pathogens: development of a disk diffusion test. *Journal of Dairy Science*.

[22] National Committee for Clinical Laboratory Standards (2002). *Development of in Vitro Susceptibility Testing Criteria and Quality Control Parameters for Veterinary Antimicrobial Agents; Approved Guideline*.

[23] Murakami K, Minamide W, Wada K, Nakamura E, Teraoka H, Watanabe S (1991). Identification of methicillin-resistant strains of staphylococci by polymerase chain reaction. *Journal of Clinical Microbiology*.

[24] Vandenesch F, Naimi T, Enright MC (2003). Community-acquired methicillin-resistant *Staphylococcus aureus* carrying Panton-Valentine leukocidin genes: worldwide emergence. *Emerging Infectious Diseases*.

[25] Poutrel B, De Crémoux R, Pillet R, Heuchel V, Ducelliez M, Rubino R (1996). Relations entre statut infectieux des mamelles et numèrations cellulaires du lait de chèvre. *Somatic Cells and Milk of Small Ruminants*.

[26] Ebrahimi A, Lotfalian S, Karimi S (2007). Drug resistance in isolated bacteria from milk of sheep and goats with subclinical mastitis in Shahrekord district. *Iranian Journal of Veterinary Research*.

[27] Moroni P, Pisoni G, Vimercati C (2005). Characterization of *Staphylococcus aureus* isolated from chronically infected dairy goats. *Journal of Dairy Science*.

[28] da Silva ER, Siqueira AP, Martins JCD, Ferreira WPB, da Silva N (2004). Identification and in vitro antimicrobial susceptibility of *Staphylococcus* species isolated from goat mastitis in the Northeast of Brazil. *Small Ruminant Research*.

[29] Alves PDD, McCulloch JA, Even S (2009). Molecular characterisation of *Staphylococcus aureus* strains isolated from small and large ruminants reveals a host rather than tissue specificity. *Veterinary Microbiology*.

[30] Georgopapadakou NH (1993). Penicillin-binding proteins and bacterial resistance to *β*-lactams. *Antimicrobial Agents and Chemotherapy*.

[31] Deinhofer M, Pernthaner A (1995). *Staphylococcus* spp. as mastitis-related pathogens in goat milk. *Veterinary Microbiology*.

[32] De Santis EPL, Virdis S, Mazzette R, Nieddu MP, Farina S, Corona A Sensibilità nei confronti degli antibiotici di stafilococchi coagulasi negativi isolati in mastiti subcliniche dell’ovino.

